# Effectiveness of two web-based cognitive bias modification interventions targeting approach and attentional bias in gambling problems: study protocol for a pilot randomised controlled trial

**DOI:** 10.1186/s13063-017-2190-2

**Published:** 2017-10-03

**Authors:** Marilisa Boffo, Ronny Willemen, Thomas Pronk, Reinout W. Wiers, Geert Dom

**Affiliations:** 1 Addiction Development and Psychopathology (ADAPT) lab, Department of Psychology, University of Amsterdam, Amsterdam, Netherlands; 2 Collaborative Antwerp Psychiatric Research Institute (CAPRI), Antwerp University (UA), Wilrijk, Belgium; 3 Centre for Alcohol and other Substance Problems (CAD Limburg), Hasselt, Belgium; 4 Department of Psychology, University of Amsterdam, Nieuwe Achtergracht 129, 1018 WS Amsterdam, The Netherlands

**Keywords:** Cognitive bias modification, Gambling, Addiction, Approach bias, Attentional bias, Randomised controlled trial, e-health

## Abstract

**Background:**

Disordered gamblers have phenotypical and pathological similarities to those with substance use disorders (SUD), including exaggerated automatic cognitive processing of motivationally salient gambling cues in the environment (i.e., *attentional* and *approach bias*). Cognitive bias modification (CBM) is a family of computerised interventions that have proved effective in successfully re-training these automatic cognitive biases in SUD. CBM interventions can, in principle, be administered online, thus showing potential of being a low-cost, low-threshold addition to conventional treatments. This paper presents the design of a pilot randomised controlled trial exploring the effectiveness of two web-based CBM interventions targeting attentional and approach bias towards gambling cues in a sample of Dutch and Belgian problematic and pathological gamblers.

**Methods/design:**

Participants (N = 182) are community-recruited adults experiencing gambling problems, who have gambled at least twice in the past 6 months and are motivated to change their gambling behaviour. After a baseline assessment session, participants are randomly assigned to one of four experimental conditions (attentional or approach bias training, or the placebo version of the two trainings) and complete six sessions of training. At baseline and before each training session, participants receive automated personalised feedback on their gambling motives and reasons to quit or reduce gambling. The post-intervention, 1-month, and 3-month follow-up assessments will examine changes in gambling behaviour, with frequency and expenditure as primary outcomes, and depressive symptoms and gambling-related attentional and approach biases as secondary outcomes. Secondary analyses will explore possible moderators (interference control capacity and trait impulsivity) and mediators (change in cognitive bias) of training effects on the primary outcomes.

**Discussion:**

This study is the first to explore the effectiveness of an online CBM intervention for gambling problems. The results of this study can be extremely valuable for developing e-health interventions for gambling problems and further understanding the role of motivational implicit cognitive processes underlying problematic gambling behaviour.

**Trial registration:**

Netherlands Trial Register, NTR5096. Registered on 11 March 2015.

**Electronic supplementary material:**

The online version of this article (doi:10.1186/s13063-017-2190-2) contains supplementary material, which is available to authorized users.

## Background

Gambling disorder (GD) is a maladaptive pattern of wagering that persists in spite of detrimental consequences on major areas of life functioning, including impairment or loss of relationships, stress-related medical problems, elevated risk of suicide, criminal offences, and financial and vocational problems [1, 2]. The prevalence of lifetime GD ranges from 0.4% to 2% in the general population, with subclinical gamblers, also referred to as problem gamblers, contributing an additional 1.3–2.3% [3]. In addition, GD is comorbid with many mental health disorders, with the highest mean prevalence of substance use disorders (SUD), depression, and anxiety disorders [2–4].

Recent findings have shown that GD and SUD share many psychopathological features, including increased impulsivity and loss of control, impairments in response inhibition, cognitive flexibility and self-regulatory behaviour; craving, tolerance, and withdrawal symptomatology; gambling-related cue reactivity and selective attention; and neglect of other areas of life [5–10]. Similar to SUD, pathological gamblers show increased reward-seeking behaviour when anticipating winning together with lower reward and punishment sensitivity after winning or losing [11–15] (for a recent review of neuropsychological and neurobiological similarities between GD and SUD, see [16–18]). The clinical and neurobiological similarities between SUD and GD and their high co-occurrence further grounded the inclusion of GD within the substance-related and addictive disorders category in the latest *Diagnostic and Statistical Manual of Mental Disorders, Fifth Edition* [1].

The categorisation of GD as a form of behavioural addiction may also relate it to recent dual-process models of addictive behaviours, which postulate the existence of two intertwined but qualitatively different cognitive processes, i.e., impulsive and reflective processes, which underlie the onset and maintenance of addictive behaviours [19–22]. Impulsive processes involve fast automatic processing of motivationally salient substance-related cues in the environment driven by enhanced implicit motivation to consume the substance and decreased motivation to engage in other activities. Reflective processes are delayed, slower control processes of behaviour and emotion regulation, which involve goal-driven monitoring and decision-making processes, expectations and the evaluation of short-term and long-term consequences of behaviour. Through repetitive experiences with the substance, gradual classical conditioning learning processes, and habit formation, substance-related stimuli and behaviours can acquire incentive salience properties for triggering impulsive, automatic, and involuntary motivational states [17, 23]. As a result, environmental substance-related cues may be flagged as motivationally salient, grab selective attention, and endow the individual with a state of preparedness and behavioural approach tendencies towards cues that signal the upcoming reward [20]. If strong enough, these processes could interfere with or disrupt higher-order cognitive and affective mechanisms, which are necessary to enact cognitive and behavioural control and enable the individual to resist the temptation to exhibit addiction-related behaviours. Similar to SUD, one of the essential features of behavioural addictions is the failure to resist an impulse, drive, temptation, or craving for something that is potentially harmful to oneself or to others. The repetitive engagement in these behaviours ultimately “hijacks” individual resources at the expense of other personal life domains.

The hypersensitisation of impulsive processes and detriment of cognitive control and emotion regulation processes can put the individual more at risk of being stirred to gamble despite the occurrence of aversive consequences for the individual’s life and environment. These conditioned impulsive processes, named *cognitive biases*, include attentional processes (e.g., the automatic tendency to selectively attend and quickly process salient cues, also referred to as *attentional bias*) and automatically triggered action tendencies towards motivationally salient cues (e.g., the automatic tendency to approach gambling games and/or sites, also referred to as *approach bias*). A few studies have explored attentional biases in problematic and pathological gamblers (for a narrative review, see Hønsi et al. [24]). Both groups generally had faster reaction times when responding to gambling-related stimuli relative to other stimulus categories [8, 9, 25–27], consistent with results from studies with substance users [28]. Further, neuroimaging studies on cue reactivity in pathological and problematic gamblers compared to healthy controls identified increased responsiveness in fronto-striatal reward circuitry and brain areas related to attentional processing of gambling stimuli [6, 29, 30]. To date, no study has yet explored the occurrence of gambling-related approach tendencies.

Novel interventions have been designed to retrain these abnormal impulsive processes through the use of cognitive bias modification paradigms (CBM). CBM interventions, such as attentional bias modification (ABM) training [31] and approach bias modification training (AppBM) [32], aim to directly target the dominant cognitive biases playing a role in disorders. CBM interventions have often employed variations of the same computerised tasks used to assess the bias, with the addition of a contingency to manipulate the bias in the desired direction (for a review, see [21]). However, non-computerized varieties have also been developed [33]. Clinical studies with substance-dependent patients showed that the addition of AppBM and ABM interventions on top of standard treatment was effective not only in reversing the approach or attentional bias immediately after the training, but also in reducing relapse rates at 1-year follow up [34, 35] or in extending time to relapse in the experimental condition [36].

The maladaptive selective information processing and conditioned mechanisms underlying problematic and pathological gambling suggest that CBM paradigms may also be effective in reversing gambling-related cognitive biases, thus potentially reducing affective reactivity and changing habitual behaviours [13, 16, 24, 37]. To date there is no study exploring the effectiveness of CBM for GD. However, a first pilot study in another form of behavioural addiction, online gaming, reported that a single session of AppBM decreased gaming-related approach bias and positively affected gaming behaviour [38].

CBM interventions can in principle be administered online, thus showing potential of being a low-threshold, low-cost addition to more conventional treatments, such as cognitive behavioural therapy and motivational interviewing. This CBM feature is of particular value for new gambling treatment venues, since only 10% of problematic and pathological gamblers seem to seek help and enter treatment [39], likely because of shame and stigma about their condition or because they are unaware, reluctant, or unavailable to start face-to-face treatment [40, 41]. Providing web-based interventions can then increase the accessibility to help-resources for gamblers by ensuring anonymity and circumventing many of the barriers associated with traditional in-person treatments. However, there are indications that online CBM interventions may be less effective than clinical interventions [42], although a recent trial in smoking cessation provided promising results [43].

The present paper reports the full design of a pilot randomised controlled trial (RCT) exploring the potential effectiveness of ABM training and AppBM training delivered online. For efficiency reasons, the two training modules are tested under the same protocol, each one compared to its own control condition, which is analogous to running two studies simultaneously. In addition to the training programme, all participants receive personalised motivational feedback on their motives to gamble and reasons to reduce or quit gambling, in order to support their compliance wth the training intervention [44] and promote the intrinsic motivation to change their gambling behaviour [45].

### The present study: objectives and hypotheses

Being the first CBM study in the gambling field, the primary goals of the RCT are to explore (a) whether gambling behaviour decreases over time as a result of a CBM intervention, and (b) whether the two CBM interventions would successfully decrease or reverse the targeted bias. Primary outcomes are monthly frequency and expenditure (i.e., average amount of money spent per month) [46], assessed at baseline, after the intervention, and in the medium term (after 3 and 6 months). The secondary outcomes include measures of gambling-related attentional and approach bias, administered at baseline, after the intervention, and at the 3-month follow up. Participants’ severity of gambling problems and depressive symptoms are also monitored over all time points.

An additional secondary moderated mediation analysis will explore the moderating effects of trait impulsivity and interference control capacity (i.e., cognitive inhibition), which have been considered as endophenotypical vulnerability markers for SUD and GD [47, 48]. Greater impulsivity and diminished ability to cope with cognitive interference could lead to lesser ability to ignore gambling cues in the environment and to suppress prepotent behavioural responses, and could thus moderate the strength of the cognitive biases. In line with previous results of CBM studies in alcohol addiction [21, 35], any change in cognitive bias as a result of the training intervention would mediate changes in the gambling outcome variables. Further, participants with stronger cognitive biases and/or lower interference control capacity and higher impulsivity at baseline would benefit more from a CBM intervention than participants with weaker cognitive biases and/or stronger interference control capacity and lower impulsivity.

## Methods

### Study design

The study is a pilot, double-blind RCT with a four-group parallel design: two groups complete either the ABM training or the AppBM training and two control groups receive the placebo version of either training. The two placebo training varieties consist of a continuous assessment task without any stimulus-response contingency (i.e., participants are equally trained away from and towards both gambling and control stimuli [34, 35, 44]). The manipulation of the task stimulus-response contingency offers greater control over the comparison between experimental and placebo conditions, ruling out non-specific effects related to performing a computerised intervention, while retaining the same tasks, stimuli, and instructions. Given the absence of previous studies on the effects of CBM interventions with gamblers, the present design does not introduce a factorial combination of the two CBM interventions, since the two training interventions have never been individually tested with gamblers before, making it premature to experimentally test any interaction effect.

Participants complete a total of 10 sessions: a first baseline assessment session including the automated personalised feedback on their reported gambling motives and beliefs, six training sessions [49], a post-intervention assessment, and two follow-up assessment sessions after 3 and 6 months. Each training session starts with shortened automated personalised feedback about participants’ reported negative consequences of their gambling and advantages of reducing or quitting, after which motivation to train and craving for gambling are assessed, and proceeds with the training task (about 15 minutes). Participants can train almost daily, with a between-session interval of at least 20 hours; however, they are suggested to complete two to three sessions per week, allowing them to complete the full intervention in about 3 weeks. The total duration of the study is estimated to be about 7.5 months for each participant. The entire study, including all assessment measures, computerised tasks and automated personalised feedback, is delivered online through the LOTUS and JASMIN experiment delivery online platforms developed and managed by the University of Amsterdam.

**Fig. 1:**
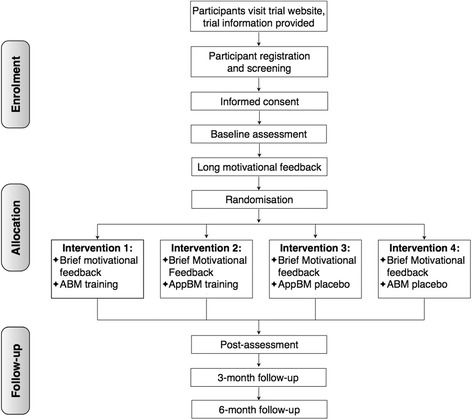
CONSORT flow diagram of participants' progress through the phases of the RCT

The CONSORT participant flowchart [50] is presented in Fig. 1. The study protocol has been reviewed and granted ethics approval by the Medical Ethics Committee of the University of Antwerp (October 2014, Belgian registration number: B300201422158) and by the Ethics Committee of the University of Amsterdam (August 2014, Protocol number: 2014-DP-3774). Any major change in design and/or experimental procedure will be submitted to the ethics committees through amendments to the protocol. Any adjustment related to technical trouble-shooting and/or maintenance of the online platform does not require amendment with the ethics committees. The study has been registered in the Netherlands Clinical Trial Registry (identifier: NTR5096), listing all items of the World Health Organization Trial Registration Data Set (see also SPIRIT checklist in Additional file 1).

### Participants and procedure

Participants are adults with gambling problems community-recruited in the Netherlands and Belgium through promotion advertisements and banners on local self-help websites for gambling problems and gambling online forums and organisations, and via flyers and promotion meetings around local addiction-care facilities. All study materials are both in Dutch/Flemish and French and participants can select their preferred language when creating their account. Participants are screened for eligibility at registration on the study platform according to the following criteria:

1. Inclusion criteria: older than 18 years, having gambled at least twice in the past 6 months, seeking help for gambling problems (intrinsic motivation to treatment);
2. Exclusion criteria: not being Dutch/Flemish or French native speaker, not having almost daily Internet access.

To encompass the full range of potential problems from problematic gambling to disordered gambling and to maximise participant inclusion, there is no inclusion criterion based on the severity of gambling problems (South Oak Gambling Scale score ≥ 3 [51]). Participants do not have any restriction in concomitant care and/or treatment while participating in the study, nor are they requested to provide information thereof. As an online self-help intervention, CBM training can be autonomously completed as an adjunct to any other type of intervention.

The use of broad eligibility criteria will ensure the inclusion of both problematic and pathological gamblers, thus not restricting the applicability of the interventions to severe gamblers only. This would also enhance the external validity of the study, since the results will be retrieved from a broad, subclinical and clinical sample of gamblers.

Upon registration, the goals, conditions, and procedure of the study are fully explained to participants, after which they provide their informed consent to participation. Participants regularly receive reminder emails about their open or upcoming training sessions and will be excluded from the study only if they do not complete the baseline assessment within 30 days after registration or if they request to be excluded. Data of excluded participants will not be included in the final analyses of the study outcomes. Participants’ data are permanently deleted from the database only upon participants’ explicit request to do so.

Once they complete the study, participants are free to enrol in a second booster training module. Participants assigned to the two placebo conditions are notified with their actual condition assignment and will randomly complete one of the two CBM programmes. Participants assigned to the active training conditions are notified of being part of the approximately 61% of participants who received one of the active interventions and will complete the training module they have not received during the experimental phase.

#### Randomisation

Upon completing the baseline assessment session, participants are automatically randomised by the computerised system into one of the four experimental conditions in two steps: first, participants are randomised into three main experimental arms (ABM, AppBM, or placebo) with a 1:1:1.25 allocation ratio. Participants assigned to the placebo condition are then further randomised to receive the placebo version of either the AppBM or the ABM training, with a final allocation ratio equal to 1:1:0.62:0.62. Both randomisation steps are stratified by gender (i.e., participants are randomly allocated to one of the conditions to which the fewest participants of their gender have been assigned so far). The two-step randomisation procedure and the uneven ratio over the four experimental conditions were designed to increase the chance of receiving at least one active training intervention, while still allowing for a univocal comparison of each active training module with their respective placebo condition. Allocation concealment is fully ensured, since participant randomisation is fully automated and performed by a computer algorithm.

#### Blinding

Since the study procedure is fully automated, with all training and assessment sessions fully delivered online, both the participants and the investigators are not aware of which experimental condition participants are assigned to. To prevent participants from guessing the training intervention they receive, they are required to respond to an irrelevant feature in all training paradigms (e.g., the orientation of the picture or probe) instead of reacting to the content of picture (gambling or non-gambling) [32, 34, 35, 44]. To also guarantee full blinding during data analyses, the dataset for the outcome analyses will be retrieved from the online platform in anonymised format only at conclusion of the data collection. Accurate storage and back-up of all collected data is independently handled by a member of the information technology (IT) department.

#### Data management

Participants create their personal research account at registration on the study online platform and can login from their own computer whenever and wherever they wish. Participant data are anonymised via the assignment of a four-digit user-ID number. Online data handling and storage conform to requirements of EU legislation on data protection: communication between project servers and devices used by participants or researchers for conducting, managing, or taking part in the study is encrypted through Secure Sockets Layer (SSL) encryption technology and databases are maintained securely behind a firewall. All core technologies, such as database, web-server, server-side and client-side frameworks are regularly inspected and updated. Sensitive data (such as participant task results) are stored in databases that are separate from those storing personally identifiable data (such as username, password, email address, if any). The two databases are running on different machines managed by different system administrators and institutions (University of Amsterdam and Qualtrics). Hence, multiple systems need to be breached in order to obtain sensitive data and link these to an individual. Finally, complete confidentiality applies to all data, and any data used for post-study analyses are anonymised.

The RCT does not include a data monitoring committee. Data collection and participants’ process along the study sessions is regularly monitored by two of the primary investigators with the support of the IT department through inspection of the activity logs on the online platform. The inspections are mainly in order to detect and solve any technical problem with the online system and monitoring participants’ inclusion rate and adherence to the study procedures. Since the launch of the study, yearly review meetings with all primary investigators have been scheduled independently from the study sponsor in order to evaluate and discuss the trial progress, with a special focus on participants’ recruitment and drop-out rates. At conclusion of the data collection, all primary investigators will have access to the final trial dataset independently from the sponsor organisation and without any type of limitation.

### Intervention

#### CBM interventions

Each training task, both in the active and placebo version, consists of four blocks: a brief practice block, a brief assessment block, and two active or placebo training blocks. The assessment block serves the purpose of measuring the strength of the bias at the start of every session and tracking any progressive change in the cognitive bias as a result of the CBM training.

In each task, each trial starts with a fixation cross in the centre of the screen for a duration randomly drawn from a uniform distribution ranging between 500 ms and 1000 ms. This setting is designed to keep participants’ attention focused and to avoid anticipatory responses. Whenever a wrong response is given, a red cross appears on the screen to allow for correction. The inter-trial interval is 500 ms. Both active and placebo training modules present a total of 2016 training trials divided into 6 sessions (336 training trials per session).

Stimuli presented in the assessment and training tasks are tailored to participants’ choice of gambling categories. At baseline, participants are asked to choose two gambling activities they feel are or could be the most problematic out of five categories (roulette and dies, slot machines, card games, sport betting, and Belgian bingo). Multiple random samples of stimuli for the two selected gambling activities and respective control pictures will then be presented in all tasks. Practice blocks present neutral stimuli (grey geometrical pictures) to practice the task instructions.

#### Attentional bias training

Attentional bias is assessed and trained through an adapted version of the Visual Probe Task (VPT) [31, 36, 44]. The VPT is a computerized speeded reaction-time task in which participants are asked to respond to probes located in two different positions on the computer screen. During the task, a gambling-related picture and a control picture are presented next to each other on the screen for 500 ms. After the stimuli presentation, a small arrow (8.3% of the width/height of the picture) pointing upwards or downwards is presented for 750 ms in either of two trial formats: it replaces one of the two pictures (*after* format) – measuring speeded detection of gambling-related stimuli – or is positioned on top of one of the pictures (*on top* format) – measuring the relative difficulty to disengage from gambling-related stimuli. Assessment trials in the training sessions are presented in a between-session alternating block design, with trials for each format presented in separate blocks in separate sessions; whereas the two trial formats are intermixed in the training blocks. Participants are instructed to respond as fast as possible to the direction of the arrow, by pressing the corresponding key on the keyboard (U and N). The response window is set to 4000 ms. In case of no response the trial is restarted after repeating the task instructions.

The assessment version of the task is composed of three blocks: one practice block and two test blocks, one per trial format, presented in counterbalanced order across participants. The arrow is presented on the gambling picture (gambling trials) and the control picture (non-gambling trials) equally often. Attentional bias is computed by subtracting the median response time (RT) for correct responses on gambling trials from the median RT for correct responses on non-gambling trials, separately for the two trial formats. In the training version of the task, participants in the active condition are trained to direct their attention away from gambling cues and towards neutral cues by exposing them only to non-gambling trials, whereas participants in the placebo condition are presented with 50% gambling and 50% non-gambling trials (as in the assessment version).

Stimuli are pairs of matched gambling and non-gambling pictures, which are counterbalanced with a 2 × 2 design in assessment and placebo training blocks (stimulus position on the screen: left or right; arrow location: on the gambling or on the non-gambling picture) and counterbalanced only for stimulus position in active training blocks. Probe direction is set randomly upwards or downwards with the restriction that up and down appears equally often.

#### Approach bias training

Automatic approach tendencies towards gambling are assessed and trained with the modified Approach-Avoidance Task (AAT), including a zooming feature [32, 34, 35, 44, 52]. The AAT is a computerized speeded reaction-time task in which participants are asked to react to the stimulus presentation format and ignore the stimulus content.

In this task, a gambling-related picture or a control picture is presented at the centre of the screen. The picture is tilted five degrees to the left or to the right. Participants are instructed to respond to the tilt direction of the picture by pushing pictures tilted to the left away and pulling pictures tilted to the right closer. The combination of tilt direction and response (left/push and right/pull versus left/pull and right/push) is counterbalanced across participants. In order to perceptually mimic the approach/avoidance effect, a zooming effect progressively decreases the picture size upon a push response, whereas it increases it upon a pull response. The average zooming duration is 750 ms; the stimulus stays on screen for 3000 ms; in the case of no response the trial is re-started after repeating the task instructions.

The assessment version of the task is composed of three blocks: one practice block and two test blocks. Gambling and control pictures are presented equally often in both push and pull formats. Approach bias scores are computed by subtracting median RTs for correct responses to pull and push trials for each stimulus category: (gambling/push – gambling/pull) and (control/push – control/pull). For each stimulus category, a positive score indicates relatively faster RTs for approach responses compared to avoidance, whereas a negative score indicates relatively faster RTs for avoidance responses compared to approach. In the active training version, participants are trained to avoid gambling cues by exposing them only to gambling/push and control/pull trials, whereas in the placebo version both stimulus categories are presented equally often in both formats (as in the assessment version). The stimuli are pairs of matched gambling and non-gambling pictures, which are counterbalanced for presentation format only in the assessment version.

#### Task stimuli

An ad-hoc large stimulus set was purposely created by adapting existing stimulus development guidelines for cognitive bias research to the context of gambling [53] and according to previous studies assessing gambling cue reactivity [6]. Stimuli are 40 pairs of matched pictures (500 × 500 pixels) of gambling cues and controls for each of five gambling categories: roulettes and dies, slot machines, card games, sport betting, and Belgian bingo (applicable to Belgian participants only). Pictures include common gambling games present in both the Netherlands and Belgium.

All gambling pictures were photographed in real-life gambling settings (i.e., casinos, betting rooms, and slot machine halls) with a high-resolution camera, by consistently using the same framing and shooting angle (i.e., from the front) and without using flash. Control pictures have been similarly developed and are as similar as possible in complexity and pictorial features (i.e., colour, luminosity, and shooting angle) to gambling pictures. Gambling and control pictures were matched for complexity and comparability as follows [6]: equal number of overview and detail pictures and screenshots of websites when the gambling category also included online gambling sites (i.e., sport betting, slot machines, card games, and roulette and dies). Control pictures include real-life, daily objects (or websites for online stimuli) or locations completely unrelated to gambling (i.e., no reference to money or cash dispensers, no video games or anything else connected to gambling). All pictures were processed in Photoshop CS6 (Adobe Systems Incorporated, San Jose, CA, USA) to adjust for minor imperfections, size, exposure, brightness, and contrast, and to ensure maximal picture similarity.

#### Personalised motivational feedback

Although successful in reversing maladaptive cognitive biases, online CBM interventions suffer from issues that are common to online self-help tools, namely, impersonality and large dropout rates, which, among other reasons, are possibly due to the progressive decrease of users’ engagement with the online tool [42]. Hence, the main goal of adding personalised feedback is to promote participants’ retention to the intervention, by increasing their adherence to the training intervention and supporting their motivation to change their gambling behaviour.

The content and style of the feedback draws on motivational interviewing (MI) principles [54] and combines cognitive, behavioural, and motivational elements into a focused psycho-educational leaflet, which can also be downloaded. MI is a client-centred counselling approach aimed at promoting and enhancing intrinsic motivation to change by exploring participants’ motives and pros and cons of changing their behaviour, resolving any ambivalence about change, and reinforcing their self-efficacy [54]. First face-to-face brief MI-based interventions for excessive gambling behaviour have been shown to significantly reduce gambling frequency and expenditure (for a recent meta-analysis, see Yakovenko et al. [55]). A recent clinical trial tested the effectiveness of an online version of MI-based personalised feedback on gambling behaviour, related cognitive distortions and negative consequences of gambling, and found a significant reduction in gambling frequency at follow up [45]. Importantly, also a brief advice intervention incorporating minimal MI elements (i.e., personalised feedback about one’s gambling and pros and cons of gambling, change plan worksheet) proved to be beneficial [56].

In the current study, the automated personalised feedback takes the form of a summative feedback adapted from a self-help book for problem gamblers [57], elaborating participants’ responses to an ad-hoc baseline questionnaire. The questionnaire asks about participants’ expectations and motives for gambling, any experienced negative consequence of their gambling behaviour, and the reasons for changing their gambling behaviour. The feedback is given in two stages: during the baseline assessment and during the training sessions. Immediately after completing the baseline questionnaires, the first part of the feedback thoroughly explores the participants’ reported gambling motives and positive expectancies. At the start of each training session, a more concise feedback reviews the reported negative consequences of gambling and the reasons for reducing or stopping it. When the goal is not abstaining but reducing gambling, the feedback ends with a reminder of the gambling limit plan that participants scheduled when filling in the personalised feedback questionnaire (i.e., maximum amount of time and money to be spent on gambling per day in the coming 2 weeks).

The feedback generally addresses the participant in a non-judgmental, personal, and empathic style and starts at baseline with a factual summary for each gambling motive. The feedback then continues by introducing doubt about gambling-related distortions (e.g., illusion of control) and by examining their plausibility and veracity, with the goal of highlighting the discrepancy between distorted beliefs and attitudes and actual consequences of gambling behaviour. Participants are encouraged to consider the feedback in light of their personal goals and experiences and are further provided with examples of alternative sources of reward, by suggesting action-oriented strategies to increase alternative behavioural reinforcement (i.e., resuming gambling-free lifestyle, engaging in activities incompatible with gambling, and planning leisure activities) and to strengthen social reinforcement (i.e., renewal of supportive social relationships, repairing damaged relationships, and socializing with non-gamblers).

The feedback provided at the start of each training session reminds the participants of the negative consequences they experienced because of their gambling and further reviews the chosen reasons for either reducing or abstaining from gambling. The feedback highlights personal and concrete core values (e.g., healthy relationships and financial responsibility) and reinforces self-efficacy and commitment to change behaviour to achieve the desired goal (e.g., feeling better, improving relationships, and keeping a job position).

### Measures

**Fig. 2:**
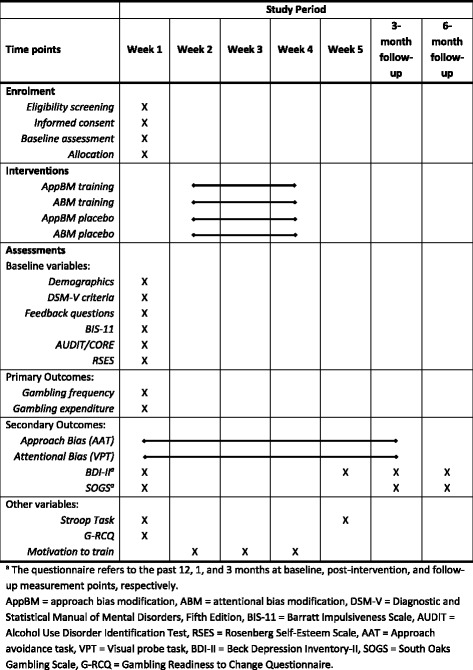
SPIRIT Figure: Schedule of forms and procedures per study time point

The schedule of all forms and study procedures along the study time points is presented in Fig. 2.

#### Baseline assessment

After registration, participants’ general socio-demographic details are collected (age, gender, highest education degree attained, personal and household monthly net income, and marital status), followed by a questionnaire asking about their habitual gambling behaviour [58]: gambling frequency in the past 12 months, in the past month and on average per month; average amount of time spent on gambling per day; average amount of money spent in the past 12 months, in the past month and on average per month; age of gambling onset (age when they gambled for the first time); any previous treatment for gambling and/or for other addictions or psychiatric disorders such as depression. Severity of gambling problems in the past 12 months (South Oaks Gambling Scale, SOGS [59]), severity of gambling addiction (itemisation of DSM-V diagnostic criteria), and preferred gambling activities are also assessed. Participants then complete the ad-hoc questionnaire about gambling motives, negative consequences, and advantages of stopping/reducing gambling.

After the personalised feedback questionnaire, participants complete a second battery of questionnaires evaluating their readiness to change their gambling behaviour (Gambling Readiness for Change Questionnaire (G-RCQ) [58]), depressive symptoms (Beck Depression Inventory-II, short version (BDI-II) [60]), substance use (CORE questionnaire short version; CORE Institute, http://core.siu.edu/surveys/index.php), and alcohol problems (Alcohol Use Disorders Identification Test (AUDIT) [61]), impulsivity (Barratt Impulsiveness Scale (BIS-11) [62]), and self-esteem (Rosenberg Self-esteem Scale (RSES) [63]).

After the questionnaires, a computerised version of the classical Stroop Task [64] is used to assess interference control capacity. In this task, participants have to classify words and symbols according to their ink colour and ignore the content. The task has been designed similar to the method used by Boffo et al. (2015) [44]. Finally, the baseline session ends with the assessment versions of the VPT and AAT, presented in counterbalanced order between participants, followed by two questions to control the quality of collected data (“While you were working on the TOP-training/your session of training, how often did things happen to distract you?”, “How much attention did you pay to the questions and/or tasks and your responses?” [65]).

#### Outcome measures

Primary outcome measures are changes in the past-month gambling frequency and expenditure (i.e., money spent on gambling, irrespective of winnings) as assessed at baseline, post-intervention, and both follow-up sessions. The secondary outcome measures include changes in attentional (VPT) and approach (AAT) bias scores from baseline to the 3-month follow-up. Changes in depressive symptoms (BDI-II) and severity of gambling problems (SOGS) from baseline to the follow-up sessions are also explored, although not included as main outcomes. Intervention credibility and expectancies are also assessed with the Credibility/Expectancy Questionnaire [66] during the second session of training, to evaluate participants’ general experience with the study.

### Data analysis

Multiple imputation of missing data will be performed for intention-to-treat analyses. Before running all analyses, all variable distributions will be screened for normality assumptions and univariate outliers. The use of parametric or non-parametric statistical tests will be adjusted accordingly. Each training group will be compared to its control group to check for any baseline difference in demographics (e.g., age and gender), severity of gambling problems (SOGS), and alcohol use. When significant differences are found and the relevant baseline variables also correlate with the outcomes of interest, they will be added as covariates in subsequent analyses.

Changes in gambling frequency and expenditure from baseline to the 6-month follow up will be examined through two separate 2 (condition: active training vs. placebo) × 4 (time: baseline, post-intervention, 3-month and 6-month follow up) mixed analysis of variance (ANOVA) analyses, one per training module. Each CBM condition is contrasted against its control condition, with the baseline assessment as reference time point for the following time points.

Changes in gambling cognitive biases as a result of each CBM intervention will be explored with a 3 (time: baseline, post-intervention, 3-month follow up) × 2 (condition: active vs. placebo training) × 2 (stimulus category: gambling vs. control) mixed ANOVA for approach bias (i.e., AAT task) and a 3 (time: baseline, post-intervention, 3-month follow up) × 2 (condition: active vs. placebo training) × 2 (trial type: on top vs. after) mixed ANOVA for attentional bias (i.e., VPT task). Any change in depression symptoms and gambling problems would be explored using two 2 (condition: active vs. placebo training) × 4 (time: baseline, post-intervention, 3-month and 6-month follow-up) mixed ANOVA analyses, one per training module.

No multiplicity adjustment will be applied to correct for the multiple treatment arms since the two active training conditions are distinct treatments, compared to their own control condition. As mentioned earlier, in order to efficiently explore the main effects of both types of training modules, the design of the study involves running two clinical trials under the same protocol. Hence, the two trials can be considered as separate studies, making it unconventional to apply any adjustments to alpha levels. Furthermore, we emphasize that the study is strictly explorative in nature, so any significant findings should be received with some reservation until tested in further confirmatory RCTs, implying a lesser need for multiple-testing correction [67].

If significant effects of the CBM interventions on the cognitive bias and gambling outcomes are detected, a moderated mediation analysis for each CBM module will be carried out to explore the moderating effect of interference control capacity, impulsivity and baseline cognitive bias on the relation between CBM effects and the gambling outcome(s) [68]. Baseline cognitive biases, interference control capacity (Stroop task) and trait impulsivity (BIS-11) would be tested as moderators of the changes in cognitive bias resulting from training, which would further act as mediator of the change in the primary outcome(s).

Besides monitoring participant inclusion and drop-out, the analysis plan does not involve any interim analysis of collected data. The clinical trial will continue until the target sample size is reached unless participant attrition rate, defined as a drop-out at any moment after completing the baseline assessment, exceeds the expected 60% attrition rate of included participants [42]. The results of the study will be reported following the guidelines of the “Consolidated standards of reporting trials” (CONSORT) extension to non-pharmacological interventions [69].

### Sample size

A conservative a priori power analysis (G*Power 3.1, open-source software [70]) required a sample size of 114 participants to detect a small-to-medium effect size (Cohen’s *f* = 0.15, equivalent to partial eta squared value of 0.02) for the interaction effect between time and experimental condition for each primary outcome, using mixed ANOVA. Power of 0.80, Bonferroni-adjusted type-I error probability of 0.025 (0.05/2 primary outcomes) and moderate correlation between the repeated measures of 0.3 were assumed. Based on a similar online CBM study for alcohol drinking problems [42], an additional 60% dropout rate at follow up was taken into account, leading to a final target sample size of 182 participants.

## Discussion

The goal of this pilot, double-blind RCT is to develop and explore the effectiveness of two online CBM interventions targeting maladaptive implicit motivational processes underlying problematic and pathological gambling behaviour, namely selective attention and automatic approach tendencies towards gambling cues. To the best of our knowledge, this is the first study evaluating the potential effects of CBM interventions on gambling behaviour. The results would expand previous research on biased information processing of gambling cues and reward-related sensitisation mechanisms, by identifying whether they are susceptible to change and generate hypotheses to be tested in further clinical studies.

CBM has been demonstrated to successfully reduce the coding of alcohol-related salient cues in the brain reward system [71] and impact on addiction behavioural outcomes [34–36]. Cognitive profiling of pathological gamblers identified deficits in response inhibition to motivationally salient cues, reward-related switching, and value-based decision-making processes [37]. Therefore, it seems reasonable to hypothesise that interventions targeting value-based and implicitly learnt stimulus-response associations could positively impact the “imbalance” in the value system and likely reduce the risk to relapse and/or escalating in gambling behaviour. Furthermore, CBM interventions are simple, inexpensive, and easy to access anywhere and anytime via the Internet, thus offering a larger outreach and greater availability, convenience, and accessibility, than standard face-to-face interventions. These aspects are important to clinical practice and for healthcare policy makers, since problematic and pathological gamblers are a notoriously difficult population to reach, who hardly ever seek help through standard healthcare facilities and resources, and when they do, the majority of gamblers drop out of treatment. Brief e-health interventions have the potential to be a valuable and cost-effective solution, accommodating the needs and characteristics of this particular population.

An innovative feature of the CBM interventions presented here concerns the introduction of some degree of personalisation in the selection of the stimuli used in the training tasks. The choice of task stimuli for both assessment and training is a key element in CBM interventions. Stimuli should be easily recognisable, sufficiently diverse in content, and representative of the environmental cues and behavioural patterns associated with the addictive behaviour of interest [53]. To this end, a substantial number of stimuli encompassing four broad categories of gambling games, plus an additional category for a local gambling practice (i.e., Belgian bingo), have been created to maximise generalisability of training effects on diverse gambling cues and scenarios [34, 35]. The gambling stimuli were developed according to a validated CBM stimulus development protocol [53] and previous studies on gambling cue reactivity [6]. Within each gambling category, pictures portray several gambling contexts, circumstances, and objects, and typical gambling venues and exemplars of popular gambling websites in Belgium and the Netherlands. However, gambling practices and instances are so numerous and heterogeneous, that using such a variety of stimuli would not properly match the gambling habits of different people. For example, one participant might regularly play poker and dice, but never bet on horse races or play slot machines. Incentive-motivational models of addiction argue that biased motivational cognitive processes such as attentional bias and approach bias are proportional to the learned association between specific gambling cues and the resulting rewarding effects [23]. Therefore, cognitive biases will be only exhibited towards cues consistently associated with rewarding effects, and not towards cues that participants have limited or aversive experiences with. Similar to previous studies evaluating stimulus-specific attentional bias towards alcohol [72–74], in the current study participants choose two gambling activities that are most problematic or could become a problem. Personalising the stimuli used in CBM tasks may thus optimise the effects of the training intervention and also improve the construct validity of the assessment tasks.

To conclude, this is the first study exploring, with a personalized approach, the effects of two online CBM interventions for individuals with different degrees of gambling problems. Results will allow further exploration of the underlying theoretical assumptions of CBM interventions, i.e., dual-process models of addictive behaviours, and their applicability to gambling disorder. In addition, results will provide preliminary data on both the implementation feasibility and effectiveness of a new treatment approach, which, also from a health-economic perspective, might be highly interesting.

## Trial status

Recruitment is open and data collection is currently ongoing (started in February 2015). We expect it to be completed at the end of 2017.

## Additional file

Additional file 1: SPIRIT 2013 checklist. (DOC 122 kb)

## Abbreviations

AAT: Approach avoidance task
ABM: Attentional bias modification
ANOVA: Analysis of variance
AppBM: Approach bias modification
AUDIT: Alcohol Use Disorder Identification Test
BDI-II: Beck Depression Inventory-II
BIS-11: Barratt Impulsiveness Scale
CBM: Cognitive bias modification
GD: Gambling disorder
G-RCQ: Gambling Readiness to Change questionnaire
MI: Motivational interviewing
RCT: Randomized controlled trial
RSES: Rosenberg Self-Esteem Scale
RT: Reaction time
SOGS: South Oaks Gambling Scale
SPIRIT: Standard protocol items: recommendation for interventional trials
SUD: Substance abuse disorders
VPT: Visual probe task
